# Small Samples, Big Problems, Statistical Tests in Nematology Research Need Power

**DOI:** 10.2478/jofnem-2025-0062

**Published:** 2026-02-02

**Authors:** Itsuhiro Ko, David Rice

**Affiliations:** Department of Plant Pathology, Washington State University, Pullman, WA 99164; Program of Molecular Plant Sciences, Washington State University, Pullman, WA 99164; Department of Mathematics and Statistics, Washington State University, Pullman, WA 99164

**Keywords:** Effect size, method, power analysis, sample size, statistical significance

## Abstract

In nematology research, hypothesis testing is a fundamental method and is typically supported by statistical significance (e.g., *P*-value <0.05). However, our review of recent publications in nematology reveals frequent issues, including unjustified sample size and unclear reporting of statistical methods, which undermines the validity and reproducibility of the results. To address these issues, we recommend researchers to conduct a priori power analyses to estimate adequate sample sizes and report key descriptive statistics (e.g., effect size). These practices not only strengthen the reliability of research, but can also help answer a central question for investigators: How many samples are needed to detect a “truly” statistically significant difference in an experiment?

Nematology studies frequently use statistical tests to evaluate differences in observable traits such as nematode mortality, reproduction factor, body size, and disease severity. These measurements are typically collected in a laboratory, greenhouse, or field, and they are used to represent the broader populations. The data are analyzed using hypothesis testing with statistical significance usually defined as a *P*-value <0.05. Despite the robustness of the hypothesis testing and *P*-values, their widespread usage without the careful consideration of their pitfalls, assumptions, and limitations may compromise the validity and reproducibility of the research findings.

Upon reviewing recent publications on plant-parasitic nematode–plant interaction publications, two common problems were identified:

1. Standard statistical testing was conducted on experiments with small sample sizes *n* = 5, which can bias representation of the population and reduce the precision and reliability of statistical tests.
2. Measures of variability, such as standard deviations (SD) or standard errors (SE), were omitted or not reported clearly, which reduced data interpretability.

We evaluated the most recent issues of the *Journal of Nematology* – Volume 56 (2024) and Volume 57 (2025). At the time of writing, it was found that of the 38 papers that used statistical tests, 17 (44.7%) used sample sizes ≤5, 20 (52.6%) did not clearly indicate whether reported variability was SD or SE (in figures, tables, or text), and 10 (26.3%) exhibited both issues.

Statistical power, which is defined as the probability of detecting a true effect (or true difference), can be an important tool to identify underpowered studies, yet it is often overlooked (Krzywinski and Altman, 2013). Conducting power analysis is recommended during the experimental design phase to help ensure that sample sizes are adequate to detect meaningful effects, reduce the risk of non-reproducible findings, and appropriately reject the null hypothesis. When statistical power is low, the likelihood of obtaining non-reproducible results increases, even when statistical standard tests (e.g., Student’s t-test or analysis of variance [ANOVA]) yield significant *P*-values. Because the *Journal of Nematology* requires that published data be reproducible, performing a power analysis is an important step to help to ensure statistical reproducibility of research.

This short commentary is designed to assist researchers who may not have a strong statistical background. It will introduce power analysis and present an example of how small sample sizes can undermine research reproducibility in nematode experiments. Lastly, we provide general recommendations for designing experiments and reporting statistically rigorous and reproducible results. We are using plant nematology studies as the primary example, but these recommendations are generally applicable across nematology.

## Power analysis in hypothesis testing

**Figure 1:**
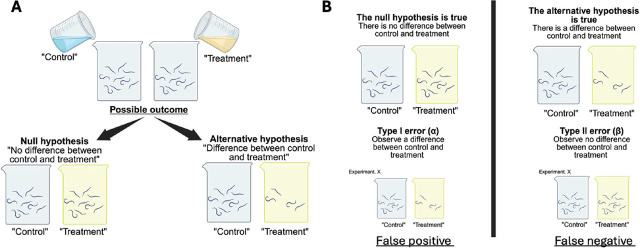
Illustration of hypothesis testing the effect of a treatment on nematode survival rate **(A)** and two types of errors that could occur **(B)**. Figure created with Biorender.com.

Statistical tests help researchers decide whether to reject the null hypothesis or not. However, these decisions are subject to two types of errors (Fig. 1). Type I error (α) is the probability of incorrectly rejecting the null hypothesis when there is truly no difference (i.e., a false positive). Researchers typically set α at 0.05, meaning they accept a 5% risk of a false positive result. A result with a *P*-value smaller than α is called “statistically significant”, leading to the rejection of the null hypothesis. The second is Type II error (β), which is the probability of failing to reject a false null hypothesis and concluding that there is no difference when one actually exists (i.e., a false negative). Because significance is often emphasized, it is easy to celebrate a single result with *P* < 0.05 while overlooking whether that finding would remain significant if the experiment were repeated. This issue can be addressed by statistical power, which represents the probability of correctly detecting a true effect and is calculated as 1-β.

The power analysis helps determine the likelihood of reproducing the same significant result in an independent experiment. A commonly used threshold is power = 0.8, which corresponds to a 20% chance of a false negative.

Assuming the data are normally distributed, statistical power is driven by (i) Sample size (*n*); (ii) effect size (a standardized difference between groups); (iii). significance level (α); and (iv). variance. Since effect size and variability are largely dictated by biological background, and α is often set by convention, planning an adequate sample size is the main lever to design “statistically powerful” experiments by researchers. Thus, power analysis is used during experimental design to determine the minimum sample size needed to detect a reproducible significant difference with a target power (e.g., 0.80).

### Example of how a small sample size affects the reproducibility of statistical results

**Figure 2:**
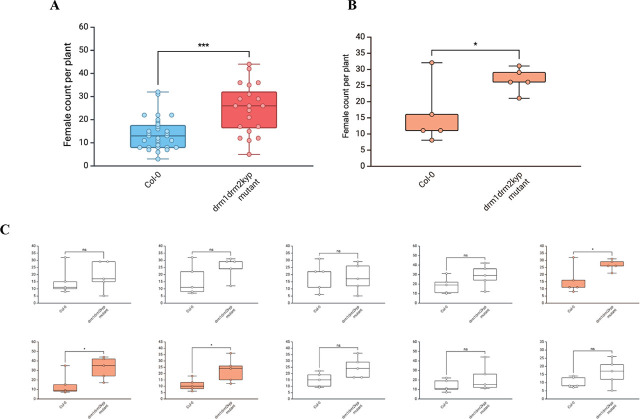
**(A)** Comparison of *H. schachtii* female counts per plant on wild-type (Col-0) and a DNA methylation-deficient triple mutant (*drm1 drm2 kyp*) *A. thaliana* (Ko et al., 2024) (Col-0: *n* = 31; *drm1 drm2 kyp*: *n* = 19). The group differences were assessed with a two-sided Wilcoxon rank-sum test. The effect size (Hedges’ *g* corrected) is 1.23. **(B)** Demo dataset created by randomly selecting five observations per group (n = 5) from Figure 2A. Two-tails Student *t-*test indicated a significant difference between two groups. **(C)** Ten simulated experiments, each with *n* = 5 per group, subsampled from the (Fig. 2A) dataset. Using a two-tailed Student’s *t*-test, statistically significant differences were observed in around 31% of simulations (orange panels). Open circles denote individual plants. The box-and-whisker plots show the median and interquartile range; whiskers indicate the full data range (min–max). Each point denotes one infected plant. Asterisks denote the group differences (^*^P < 0.05, ^***^P < 0.001).

To illustrate how sample size can influence statistical conclusions, we reanalyzed a published dataset evaluating the susceptibility of hypomethylated *Arabidopsis thaliana* mutant line (drm1drm2kyp) to the sugar beet cyst nematode *Heterodera schachtii* (Ko et al., 2024). Based on the total number of female nematodes per plant, it was concluded that the *drm1 drm2 kyp* mutant plants are significantly more susceptible than wild-type (Col-0) *Arabidopsis* (Fig. 2A)

A demonstration dataset was created by randomly selecting five observations from each group in the original study (Fig. 2B). A two-tailed Student’s *t*-test was used to compare the mean numbers of female cyst nematodes between groups as we assume data normality. The data now show a statistically significant difference in female cyst nematode numbers between two groups, rejecting the null hypothesis.

However, when we repeated this sampling procedure 10,000 times and performed a two-tailed Student’s *t*-test at each iteration, only 3,109 iterations (≈31.1%) showed that the *drm1 drm2 kyp* mutant had significantly more cyst nematodes per plant than the wild-type (Fig. 2C). In other words, the significant result in Figure 2B would be reproduced only about one-third of the time if another researcher repeated the experiment with *n* = 5 per group. The frequent false negative iterations reflect an underpowered experimental design: with a small sample size, a “significant” *P*-value is not often observed, and true differences may go undetected. To consistently detect real differences, an a priori power analysis can be used to determine an appropriate sample size. For a normally distributed dataset with equal sample size, the Cohen’s *d* effect size can be calculated using sample means (X) and pooled SD (SD_pooled_) from female nematode count of wild-type (w) and methylation mutant (m) plants (Fig. 2B):

$$\text{Cohen's}\;d\;\text{effect}\;\text{size}\left(d\right)=\frac{\left|X_{w}-X_{m}\right|}{SD_{\text{pooled}}}$$

The effect size estimated from the demo dataset (Fig. 2B) is 1.51. With *n* = 5 per group and α = 0.05 (two-tailed), the estimated power is approximately 0.55. To reach the conventional target of power = 0.80, the experiment would require at least eight samples per group. In other words, repeating the experiment with *n* = 8 per group would have about an 80% chance of detecting a significant difference between the wild-type and mutant plants, assuming the data are normally distributed.

**Figure 3:**
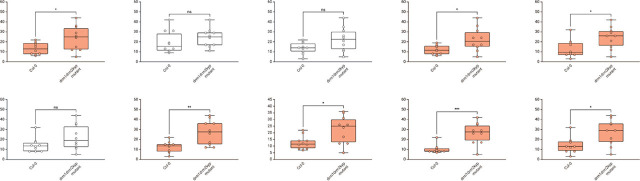
Ten simulated experiments, each with *n* = 10 per group, subsampled from the (Fig. 2) dataset. Open circles denote individual plants. Using a two-tailed Student’s *t*-test, statistically significant differences (marked with asterisks) were observed in around 70% of simulations (orange panels; ^*^P < 0.05, ^**^P < 0.01, ^***^P < 0.001).

Based on the sample size assessment, we conducted a simulation in which *n* = 10 observations were uniformly sampled per group and analyzed with a Student’s *t*-test. Repeating this procedure 10,000 times resulted in statistically significant differences in 70.1% of iterations (Fig. 3).

While effect size reflects the magnitude of difference between groups, sample size can determine how reliable an effect (difference) can be constantly detected. Because these two factors are interdependent, the effect size can influence how many samples are required for a high power experiment. Small effect sizes will require larger number of samples to detect reproducibly significant differences, whereas large effect sizes can be detected with fewer samples. In conclusion, increasing the sample size per group increases the statistical power as well as the frequency to correctly reject the null hypothesis when a true difference exists.

## Discussion

### General suggestions to enhance result reproducibility-using power analysis to estimate sample size before conducting an experiment

Simply repeating a published experiment without considering statistical power can lead to false negatives. Therefore, we recommend performing a power analysis to determine an appropriate sample size during the experimental design stage, thereby improving the reproducibility of findings. This can be done by reviewing published studies or conducting a pilot study. Doing so can provide an estimated effect size and calculate the minimal sample size to achieve a desired power to confidently reject the null hypothesis.

Websites such as Sample Size Calculator (Kohn and Senyak, 2025) and statistical software like G*Power (Faul et al., 2007) can assist with sample size determination. We note that using large language models (LLMs) such as ChatGPT (OpenAI, 2025) for sample size calculations is convenient, but may result in hallucinations (i.e., incorrectly calculating effect size from dataset). However, these LLM tools can be useful for finding relevant formulas and methods for power analysis.

**Figure 4:**
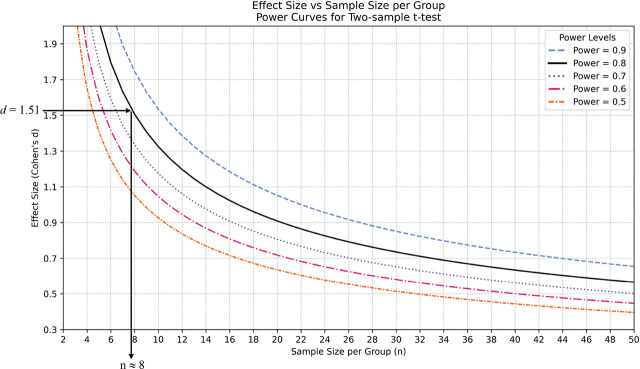
Nomogram for Cohen’s *d* effect size and power for comparing two groups of equal size using a two-sample *t-*test. Normal distributions and equal variance are assumed. Each curve represents a specific power at a significance value of α = 0.05 with a sample size on the x-axis and Cohen’s *d* effect size on the y-axis. In the example shown in Figure 3, for an experiment that has an effect size of 1.51 with a desire power of 0.8, the suggested sample size is about eight per group.

To provide researchers with a quick check on their experimental design, we created nomogram charts that can offer estimations of how many samples are required to achieve good statistical power based on estimated effect size (two sample *t-*test: Fig. 4, ANOVA: Supplementary Material 1 Figure S1 in Supplementary Materials, Maxwell et al., 1981).

Regarding a key question: what is a good power? As a rule of thumb, a power of 0.8 is the benchmark for most reproducible results (Cohen, 1988). In fields requiring stringent statistical rigor, such as medical research, higher power levels of 0.9 or above are typically mandated (Serdar et al., 2021). However, due to the high variability of nematode assays, especially in field trials with constrained sample sizes, a lower statistical power may be justifiable. It is important to remember that the statistical power of a test below 0.8 does not mean the conclusion is completely unreproducible. However, tests with power below 0.5 should be assessed with caution, as a power below 0.5 would imply <50% probability of reproducing the same results when repeating the experiment.

As a research reviewer, it is also important to know that a power analysis should not be used to judge the validity of an already published significant result. Statistic power only reflects the probability of detecting an effect in future observations; therefore, using post hoc (retrospective) power analysis to reject a published result is conceptually flawed and potentially misleading (Gent et al., 2017).

### Avoid (if possible) small sample size in an experiment

Even when an experiment yields statistically significant *P*-values, the results may not be reproducible if the study is underpowered. This is especially true when there are small sample sizes and the magnitude of the observed differences between groups is exaggerated, a phenomenon referred to as a “winner’s curse” (Button et al., 2013). Consequently, only the luckiest scientist can always observe the true difference with a small sample size (e.g., Fig. 2B).

Researchers must balance the practical considerations of their experimental design, including cost constraints and sample availability, against the expected reproducibility and credibility of their results. If the determined sample size is insufficient to meet the assumptions required for statistical testing, we recommend researchers to combine the data from multiple small-sample size experiments that share a similar setup (e.g., laboratory and greenhouse trials where most variables can be controlled). As an example, Jayasinghe et al. (2025) combined data from three independent experiments (color-coded, in their Fig. 3) before conducting a statistical test.

We recognized that field assays face inherent variability and resource limitations, which can limit their statistical power. Replications at multiple locations and years and aggregation of all data to perform statistical tests could also improve the power (Gent et al., 2017). If not possible, researchers should clearly state the effect size and can instead rely on descriptive statistics and visual representations of the data (e.g., confidence interval) and describe the trends and differences.

### Determine sample size using other approaches

As an initial step in determining the minimum sample size for a reproducible nematology experiment, power analysis can be performed under the general assumptions of the Student’s *t-*test or ANOVA, assuming data collected will be parametric with equal variance and sample size per group. When designing experiments that may involve smaller or unequal sample sizes, a corrected Cohen’s *d*, known as Hedges’ *g*, is recommended to obtain an unbiased effect size estimate for power calculations (Lakens, 2013).

Given that some data are expected to be skewed, non-normally distributed, and/or have unequal variances in field nematology studies, a Monte Carlo simulation can be used to determine a proper sample size. In this approach, a specified number of observations are repeatedly sampled with replacement from pilot experiments or published datasets. Statistical tests are then applied to each simulated dataset, and the statistical power is estimated by the proportion of iterations yielding statistically significant results at a given sample size. This procedure provides a flexible strategy for estimating sample size but requires careful specification when choosing reference datasets to perform such simulation (Gent et al., 2017; Wang and Rhemtulla, 2021).

While this paper primarily discusses the frequentist approaches that use *P-*values to compare group differences, Bayesian statistics offer an alternative way to describe such differences. Bayesian statistics consider the uncertainty of each testing group’s population parameter (i.e., mean and variance) using a distribution and evaluates how likely each parameter is, thereby allowing direct probability statements. For example, the Bayesian approach can determine the probability that, on average, there are 10 more female cyst nematodes in the hypomethylated mutants compared with wild-type plants, showing that the mutant line is more susceptible than the control. However, the Bayesian approach requires alternative procedures to determine sample size, as described in Kruschke (2013).

### Clearly report key descriptive statistics

In our survey, few publications clearly reported key statistical details such as sample size and measures of variability. Reporting power and effect sizes alongside *P*-values provides a more complete picture and helps readers assess whether a study was adequately powered to detect meaningful effects (Halsey et al., 2015). To improve the transparency and interpretability of results, we encourage researchers to report detailed descriptive statistics, including sample sizes (*n*, per group/condition), SD (or SE), effect sizes (e.g., Cohen’s *d*), confidence intervals, and, where appropriate, planned power or sample size justification. A rigorous example of preferred statistical reporting is provided in Supplementary Material 2. In addition, when experiments are repeated two or more times under the same conditions, results from all repetitions should be presented in the manuscript (or in the Supporting Materials) rather than relying on a single “representative” run.

In summary, using inappropriate statistical tests and low statistical power merely to achieve apparent significance undermines the credibility and reproducibility of experimental findings. Moreover, all authors should publish all statistical data to ensure other researchers are able to reproduce the experiments. Therefore, this commentary would encourage researchers in nematology and beyond to strengthen the reproducibility of their results by including power analysis (Supplementary Material 2). The hope is that this paper will lead to further discussion among researchers about the reproducibility of experimental results.

## Supplementary Material 1

While Cohen’s D effect size for two samples has been discussed, the *η*^2^ (Eta square) effect size is a useful effect size for one-way ANOVA. Assume a linear model:

$$y_{\mathrm{ij}}=\mu_{j}+\varepsilon_{\mathrm{ij}}$$

Where

- *j* = 1,...,*J* is the index over the treatment groups
- *i* = 1,...,*I* are the experimental units in each treatment group
- *y_ij_* is the observation index *i* for treatment index *j*
- *μ_j_* is the mean of observations in treatment group *j*
- *ɛ_i,j_* ~ *N* (0,σ^2^) are the independent, identically distributed random errors for treatment group *j* observation index *i*.

*η*^2^ can be found by calculating the ratio of the sum of square treatment (SSTr) and dividing by the sum of square error (SSError). This can be calculated using the following formula, where ${\hat{\mu }}_{j}$ is the sample mean of treatment group *j*,

$$\text{SSTr}={\sum_{j}n_{j}\left({\hat{\mu }}_{j}-\hat{\mu }\right)}^{2}$$

$$\text{SSError}=\sum_{j}\left(n_{j}-1\right)s_{j}^{2}$$

$$s_{j}^{2}=\frac{1}{n_{j}-1}\sum_{i}^{n_{j}}{\left(\hat{\mu }-y_{\mathrm{ij}}\right)}^{2}$$

$$\eta^{2}=\frac{\text{SSTr}}{\text{SSError}}$$

Where

- *n_j_* is the number of samples in treatment *j*
- $\hat{\mu }$ is the mean across all observations
- ${\hat{\mu }}_{j}$ is the mean across observations in treatment *j*
- $s_{j}^{2}$ is the sample variance for treatment *j*
- *η*^2^ is the eta squared effect size for one-way ANOVA.

While the eta square statistic is commonly reported and easy to calculate, other measures such as *ω*^2^ omega square or *f*^2^ may also be used (Maxwell et al., 1981).

We created a nomogram chart (Figure S1 in Supplementary Material) that can offer quick estimations of how many samples are required per group to achieve good statistical power based on the estimated *η*^2^ effect size, with each chart representing a different number of treatment groups, and assuming normality, equal sample size, and equal variance within each treatment group:

## Supplementary Material 2

### What should i do to strengthen research reproducibility?

***As an author analyzing data and writing a paper:***

- Report core statistics clearly in figures/tables and text: per-group sample size (*n*), what error bars represent (SD or SE), and effect size (e.g., Cohen’s *d*)
- Show all independent experiments (main or supporting materials)
- Justify sample size
- Combine identical small experiments in a single statistical analysis if small sample size is not avoidable.

A rigorous example of preferred statistical reporting and power calculation is provided below at “How to properly sample size and effect size in a paper” and “How to calculate effect size and power.”

***As a researcher who is planning an experiment:***

- Obtain effect size and variance estimates from a closely related study or a small pilot experiment.
- Use power analysis to estimate the sample sizes needed in the planned experiment, but not to assess the validity of a published result.
- Choose larger sample size for a single experiment over a small sample size for a repeated experiment.

### How to properly sample size and effect size in a paper

*******************Below is an example*******************

#### Material and Methods

The effect size in prior study (Figure 2B in the main text) was 1.51. With a significance criterion of α = 0.05 and power = 0.8, the minimum sample size per group needed with this effect size is *N* = 8 for statistical test.

**Table S1:** Descriptive statistics shown in Figure S2 in Supplementary Material

	Sample size (N)	Mean (X)	SD
Col-0 wild type (w)	10	12.3	5.56
Drm1drm2kyp mutant (m)	10	26.9	12.36

SD, standard deviation.

Therefore, in this experiment, sample size of *N* = 10 per group was obtained to test the hypothesis. …

#### Results

Col-0 *Arabidopsis* had a mean of 12.3 ± 5.56 female cyst nematodes per plant (mean ± SD), whereas the drm1 drm2 kyp knockout had 26.9 ± 12.36 per plant. A statistical test indicated that the methylation mutant was significantly more susceptible than Col-0 (*P* **<** 0.001), with an effect size of Cohen’s *d* **=** 1.52 (Figure S2 in Supplementary Material). …

*******************Above is an example*******************

### How to calculate effect size and power

Based on the mean and SD provided (Table S1 in Supplementary Material), assuming normality and equal variance, Cohen’s *D* effective size was calculated by using the below formula:

$$\text{Cohen's}\;d\;\text{effective}\;\text{size}\left(d\right)=\frac{\left|X_{w}-X_{m}\right|}{{\text{SD}}_{\text{pooled}}}$$

SD_pooled_ was calculated by using the below unweighted calculation:

$${\text{SD}}_{\text{pooled}}=\sqrt{\frac{{\mathrm{SD}}_{w}^{2}+{\mathrm{SD}}_{m}^{2}}{2}}=\sqrt{\frac{5.56^{2}+12.36^{2}}{2}}=9.59$$

Means (X) and pooled SD were then incorporated into the equation to calculate *d* and non-centrality parameter *d*:

$$d=\frac{26.9-12.3}{9.59}=1.52$$

$$\begin{array}{l} \text{Noncentrality}\;\text{parameter}\;d=\\ \frac{d}{\sqrt{\frac{1}{X_{W}}+\frac{1}{X_{M}}}}=1.52÷\sqrt{\frac{1}{10}+}\frac{1}{10}=3.40 \end{array}$$

Assuming the true non-centrally parameter is 2.49, post hoc power analysis can be performed using G*Power (Faul et al., 2007). Once opening the G*Power software, “Test family” was selected with “*t*-tests” and “Generic *t*-test” was selected for “Statistical test.” In “Type of power analysis,” “post hoc: Compute power – given a, and non-centrality parameter” was selected. Under Input Parameters, “Two tails” was selected and a “err prob” was set at 0.05. (Figure S3 in Supplementary Material) The degree of freedom (Df = 18) was calculated by the total sample number minus 2 (because of having 2 independent groups).

A power of 0.895 was achieved with an effect size (*d* = 1.52), thereby showing repeatability of this experiment.
